# Comparative Assessment of Antibiotics and Probiotics: Adjuvants in Nonsurgical Periodontal Treatment for Smokers With Generalized Periodontitis

**DOI:** 10.7759/cureus.78394

**Published:** 2025-02-02

**Authors:** Sujay Shah, Kinjal S Engineer, Rutu Shah, Hiral Shah, Shreya Gajjar, Santosh Kumar, Mainul Haque

**Affiliations:** 1 Department of Dentistry, GMERS Medical College, Gandhinagar, Gandhinagar, IND; 2 Department of Pedodontics and Preventive Dentistry, Dr. M. K. Shah Medical College and Research Center, Ahmedabad, IND; 3 Department of Anaesthesiology, GMERS Medical College, Sola, Ahmedabad, IND; 4 Department of Oral Pathology, Dr. M. K. Shah Medical College and Research Center, Ahmedabad, IND; 5 Department of Periodontology and Implantology, Karnavati School of Dentistry, Karnavati University, Gandhinagar, IND; 6 Department of Pharmacology and Therapeutics, National Defence University of Malaysia, Kuala Lumpur, MYS; 7 Department of Research, Karnavati School of Dentistry, Karnavati University, Gandhinagar, IND

**Keywords:** amoxicillin, antibiotics, colony forming units, generalized periodontitis, lactic acid bacteria, lactobacillus reuteri, metronidazole, non-surgical periodontal treatment, probiotics, scaling and root planing

## Abstract

Introduction

Humankind is suffering from the misuse and overuse of antibiotics, leading to antibiotic resistance. At this point, probiotics, if found effective, can lead to a better future. Probiotics are nothing but a colony of living-friendly bacteria. *Lactobacillus reuteri* is essential for use as a probiotic among many healthy bacteria. This study aims to compare the effectiveness of probiotics in periodontal treatment.

Methods

One hundred smokers diagnosed with chronic generalized periodontitis were randomly assigned into two groups. Group A received a seven-day course of amoxicillin 500 mg three a day and metronidazole 500 mg twice daily, followed by 30 days of probiotics, while Group B received a placebo antibiotic for seven days and 30 days of *L. reuteri* probiotics (2×10⁸ CFU daily). Periodontal parameters, including plaque index (PI), gingival index (GI), probing depth (PD), attachment loss (AL), and bleeding on probing (BOP), were utilized to asses at baseline, month one, and month one. Baseline differences were adjusted using the generalized estimating equation (GEE) model.

Results

At month three, Group B (probiotics) showed a significantly lower PI (0.52±0.49 vs. 0.88±0.52, p<0.001) and a 23% greater reduction in PD (p=0.004) compared to Group A (antibiotics + probiotics). These findings highlight the effectiveness of probiotics alone in improving periodontal health in smokers.

Conclusions

Both groups showed equal effectiveness in improving periodontal health. Hence, probiotics should be preferred over antibiotics.

## Introduction

Periodontitis is an infectious condition characterized by bacterial plaque attached to the periodontal tissues, the leading local component contributing to the disease [1]. An imbalance in the bacterial composition of the mouth's microbiota and the presence of periodontopathogens leads to dysbiosis. This can cause a chronic inflammatory response in the gum tissues, resulting in the breakdown of connective tissue and the destruction of bone tissue [2]. Diabetes and smoking are acquired or environmental risk factors that might promote and worsen the progression of periodontitis [3,4]. Smoking is a widespread and pervasive problem that impacts around 22.2% of the global population, according to the World Health Organization (2011).

Active smoking alters the immune and inflammatory mechanism of an individual's body, hence creating an adverse effect on inflammatory diseases [5]. It is often observed that smoking presents with a higher prevalence and severity of periodontitis. It exhibits greater probing depth, attachment loss, deep pockets, gingival recession, and bone loss [6].

Non-surgical periodontal treatment (NSPT) is the most preferred treatment modality for periodontal disease. NSPT includes scaling and root planing (SRP) techniques and guidance on maintaining oral hygiene [7]. The therapy is intended to eliminate the biofilm on the tooth surface, both above and below the gum line, and to prevent periodontopathogens' re-colonization of these locations [8]. The effectiveness of conventional periodontitis treatments can be affected by local or systemic factors [9]. Moreover, certain harmful microorganisms, including *Aggregatibacter actinomycetemcomitans* and *Porphyromonas gingivalis*, can infiltrate the tissues surrounding the teeth, resulting in a decreased effectiveness of this procedure in completely eradicating periodontal pathogens [10].

There is a debate regarding the impact of *Lactobacillus reuteri *and probiotics on periodontal health therapy. A 2017 review outlines the limited and temporary effects of the probiotic when used alongside SRP. The study also emphasizes the shortcomings of existing research regarding patient numbers and observation periods [11].

*L. reuteri *may have potential as a supplementary treatment during the initial phase of periodontal therapy, although the most effective dosage has yet to be established [12]. A review conducted in 2020 found limited evidence supporting the use of probiotics in reducing inflammatory markers in gingivitis. The constraints of heterogeneity and limited data are again discussed [13,14]. Ramos et al. examined the impact of systemic antibiotic and probiotic therapy (*L. reuteri*) when used alongside subgingival treatment. The study found none of these therapies provided extra advantages [15]. In their comprehensive analysis, Silva et al. examined the impact of additional probiotics on peri-implant care. They expressed dissatisfaction with insufficient evidence to support a positive outcome [16]. Ananda et al. [17] highlight the antimicrobial properties of *L. reuteri* as a supplementary treatment for periodontal disease. They also call for rigorous randomized controlled studies that focus on studying individual bacterial strains separately. Routier et al. provided an overview of the role of probiotics in future periodontal treatment, precisely when the treatment is tailored to each individual [18].

Objectives of the study

This study aims to assess the impact of administering *L. reuteri *orally as a supplementary treatment for periodontitis in smoking patients. This evaluation was conducted through longitudinal research with a short assessment period. The study is motivated by recognizing the benefits of probiotics in treating periodontitis in systemically healthy patients’ smokers. The study hypothesized that incorporating *L. reuteri* as an adjunctive therapy could enhance the treatment outcomes for generalized periodontitis in heavy smokers by positively influencing the host's immune response.

## Materials and methods

A 90-day comparative study was conducted at the Karnavati School of Dentistry in Gujarat, India. The current investigation was conducted from March 2024 to July 2024.

Selection of patients

The current study comprised 100 individuals who were heavy smokers (smoking more than 10 cigarettes per day for at least five years) and had severe generalized chronic periodontitis. Patients between the age group of 30 and 50 years diagnosed with generalized periodontitis were included as the subjects for the study. Periodontitis patients should consist of a minimum of six teeth with at least one site showing probing depth (PD) and clinical attachment loss (CAL) of 5 mm or more. Additionally, patients needed to have at least 30% of sites with PD and CAL of 4 mm or more, along with bleeding on probing (BOP). The materials used were scalers, mouth mirrors, curettes, and periodontal probes (UNC-15).

**Figure 1:**
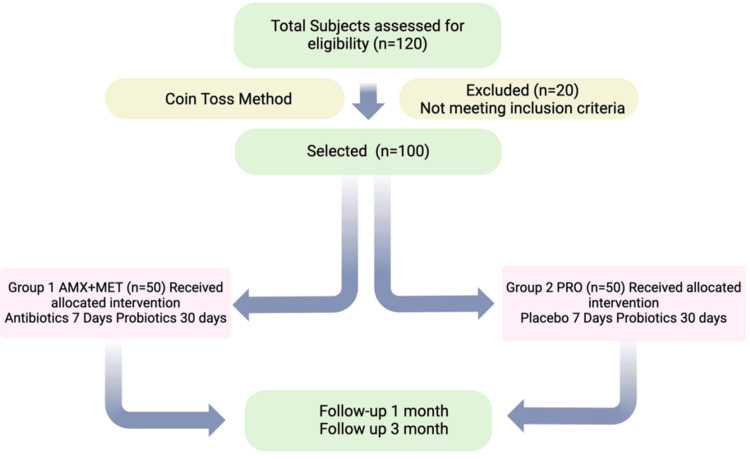
The flowchart depicting the methodology, which includes the total number of patients, final number of patients after inclusion and exclusion criteria, number of patients in each group, and follow-up periods. Notes: This figure has been drawn utilizing the premium version of Biorender [19] with the agreement license no: CX27UOB1A8, Accessed on January 30, 2025. Illustrattion Credit: Santosh Kumar

They also needed to be heavy smokers, defined as smoking more than 10 cigarettes per day for at least five years. The mouth usually comprises a minimum of 15 teeth, excluding the third molars. The exclusion criteria for this study were as follows: antibiotic use within the past six months, a history of scaling and root planing, use of medications impacting periodontal health, undergoing orthodontic treatment, the presence of any systemic condition, and pregnancy. Participants first completed a questionnaire assessing their general and oral health to determine their eligibility for inclusion. The questionnaire collected information on participants' age, smoking habits (including whether they smoked, how many cigarettes they smoked per day, and for how long they had been smoking), the presence of any systemic diseases (specifically noting whether they had diabetes and their blood pressure details), and any medications they were currently taking. Those who met the required criteria proceeded to undergo a clinical evaluation. The following flowchart illustrates the methodology of this study (Figure 1).

Clinical parameters

The clinical parameters assessed in this study included plaque index ((PI) Silness and Loe, 1964), clinical attachment level (CAL), PD, gingival index ((GI) (Loe and Silness), and BOP. The clinical examination assessed six sites per tooth throughout the mouth for PI, PD, CAL, GI, and whether BOP was present or absent. A single calibrated blinded examiner recorded all the data to reduce inter-examiner bias (one at a time). The measurements were recorded using a periodontal probe marked in millimeters (UNC-15, Hu-Friedy, Chicago, IL).

The means and standard deviations of PI, PD, and CAL were calculated from the total patient measurements across all sites to compare the therapeutic outcomes. BOP data were expressed as percentages, and the mean and standard deviation for the entire mouth were also calculated.

Furthermore, the proportion of sites with a probing depth of ≥5 mm and BOP was assessed during both periods (baseline, one month, and three months) [20]. The means and standard deviations of PD and CAL in moderate and deep pockets were analyzed and compared across the treatment groups and evaluation periods [21].

Treatment protocol

Baseline periodontal clinical examinations were conducted to assess the parameters of PI, PD, CAL, gingival recession (GR), and BOP before initiating any therapeutic intervention. Additionally, it was evaluated twice during the follow-up period at one month and three months. The patients were promptly provided comprehensive information regarding periodontal disease causes and specific recommendations for maintaining oral hygiene following the therapeutic intervention. This encompassed directions regarding toothbrushes, dental floss, and interproximal brushes that were meticulously adapted to their needs. No restriction regarding food was advised. The oral hygiene guidelines were reiterated during every visit. Participants were instructed to brush their teeth twice daily using the modified Bass technique and floss after every meal. We followed the randomization procedure (computer generated) for the current research to determine the experimental and control groups.

One hundred patients were randomized to one of the following therapy groups: Group 1 (AMX+MET) (n=50) who underwent a scaling and root planing session using ultrasound and manual curettes, along with 400 mg of metronidazole (twice daily) and 500 mg amoxicillin (thrice daily) for seven days consecutively along with 30 days probiotic placebo twice daily. Antimicrobials were selected according to the American Academy of Periodontology (AAP) for severe periodontitis. Group 2 (PRO: *L. reuteri *(DSM 17938) (n=50) underwent the same scaling and root planing session using ultrasound and manual curettes, followed by receiving the same placebo antibiotics for seven days, the probiotic *L. reuteri* (Protectis, Optibac Probiotics, Probotic Learning Lab, UK and Ireland) chewable two tablets daily for 30 days.

The probiotic tablets were carefully removed from their packaging and placed into vials labeled with a pattern matching the placebo group. Each vial contained enough tablets for the duration of our study. After brushing their teeth, the patients were instructed to consume two chewable probiotic tablets daily, one in the morning and one in the evening. This regimen should be followed for 30 days as recommended by the earlier research [22].

Patients were advised to refrain from using any product, including probiotics, antibiotics, and anti-inflammatories, for 90 days [23]. Although the vials containing probiotics and placebos were indistinguishable in appearance, the packing label clearly described them to enable professionals to differentiate them - we followed a double-blind strategy. After completing the scale procedure, subjects were promptly administered the placebo and probiotic.

Follow-up

The patients were summoned for a follow-up evaluation eight days following the phase I therapy to assess the efficacy of the treatment and identify any potential adverse effects. Participants were questioned about possible adverse effects from probiotics or placebos, including allergies, headaches, discomfort, and diarrhea. Furthermore, every patient was instructed to bring back the container of tablets given to them 31 days following the SRP to tally the remaining tablets and assess their compliance with the medication. After 90 days following periodontal therapy, the patients were contacted for a comprehensive evaluation of their entire mouth, during which probing was conducted. During these instances, the subjects were interviewed regarding modifications in their medical history, the use of concurrent medication, and any adverse effects consequences.

Statistical analysis

A one-way ANOVA model was used to estimate the mean differences in PE, GI, PD, BOP, and attachment loss between Group A and Group B. A generalized estimating equations (GEE) model with an exchangeable correlation matrix was applied to evaluate the treatment effects on outcome over time while accounting for patient autocorrelation. The GEE model was adjusted for covariates, such as age, time (at baseline, month one, and month two), and the interaction between treatment groups and time. All data analyses were conducted using Stata/IC, version 15 (Stata Corp, TX), and figures were generated with GraphPad Prism (version 8.3.2; GraphPad Software, San Diego, CA). A p-value <0.05 was considered statistically significant.

Ethical approvals

The current research was conducted from March 2024 to July 2024 and received approval from the Human Research Ethics Committee of Karnavati School of Dentistry, Gandhinagar, Gujarat, India, on 23 November 2023 (with reference no.: (KSDEC/23-24/Apr/028)).

## Results

**Figure 2:**
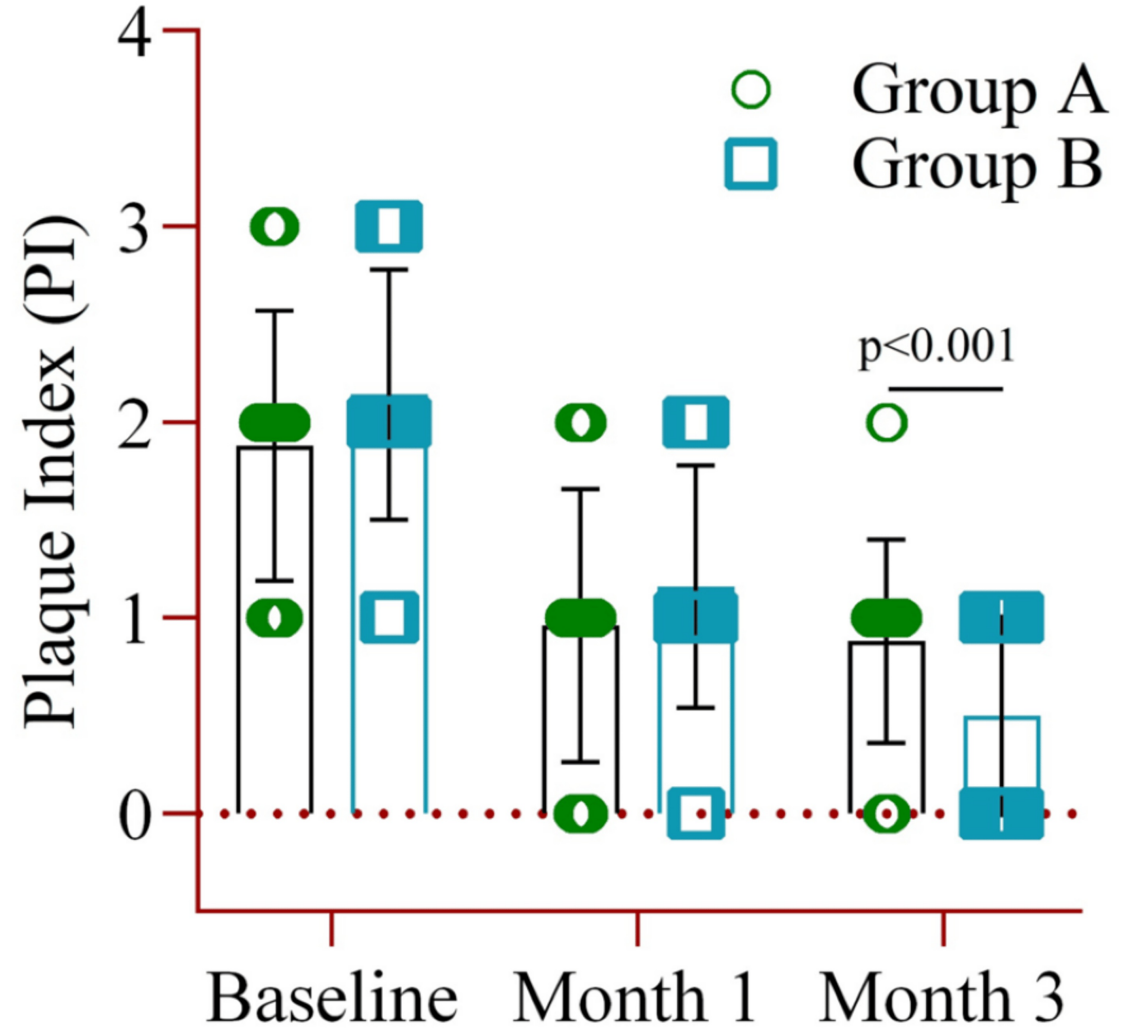
The mean difference in the plaque index between Group A (amoxicillin and metronidazole) and Group B (probiotics placebo) at baseline, month one, and month three. Notes: Two-way ANOVA was used to estimate the p-value. Illustration Credit: Md Ahsanul Haq

The PI showed no significant difference between the baseline and month one groups. However, at the end of the study, Group B demonstrated a significantly lower PI (mean±SD: 0.52±0.49) compared to Group A (0.88±0.52), with p<0.001 (Figure 2).

**Figure 3:**
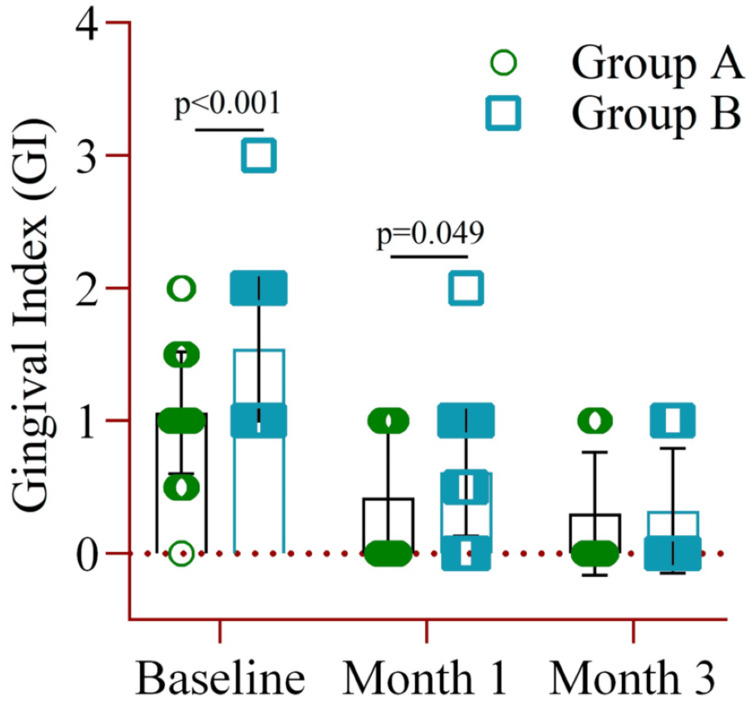
The mean difference in the gingival index between Group A (amoxicillin and metronidazole) and Group B (probiotics placebo) at baseline, month one, and month three. Notes: Two-way ANOVA was used to estimate the p-value. Illustration Credit: Md Ahsanul Haq

A comparison of the mean GI between the two groups revealed that, at baseline, Group A had a significantly higher index (1.54±0.46) compared to Group B (1.06±0.54). A similar significant difference (p=0.049) was observed at month one, with Group A showing a mean±SD of 0.42±0.49 and Group B showing 0.61±0.48. However, no significant difference was noted between the groups in month three (Figure 3).

**Figure 4:**
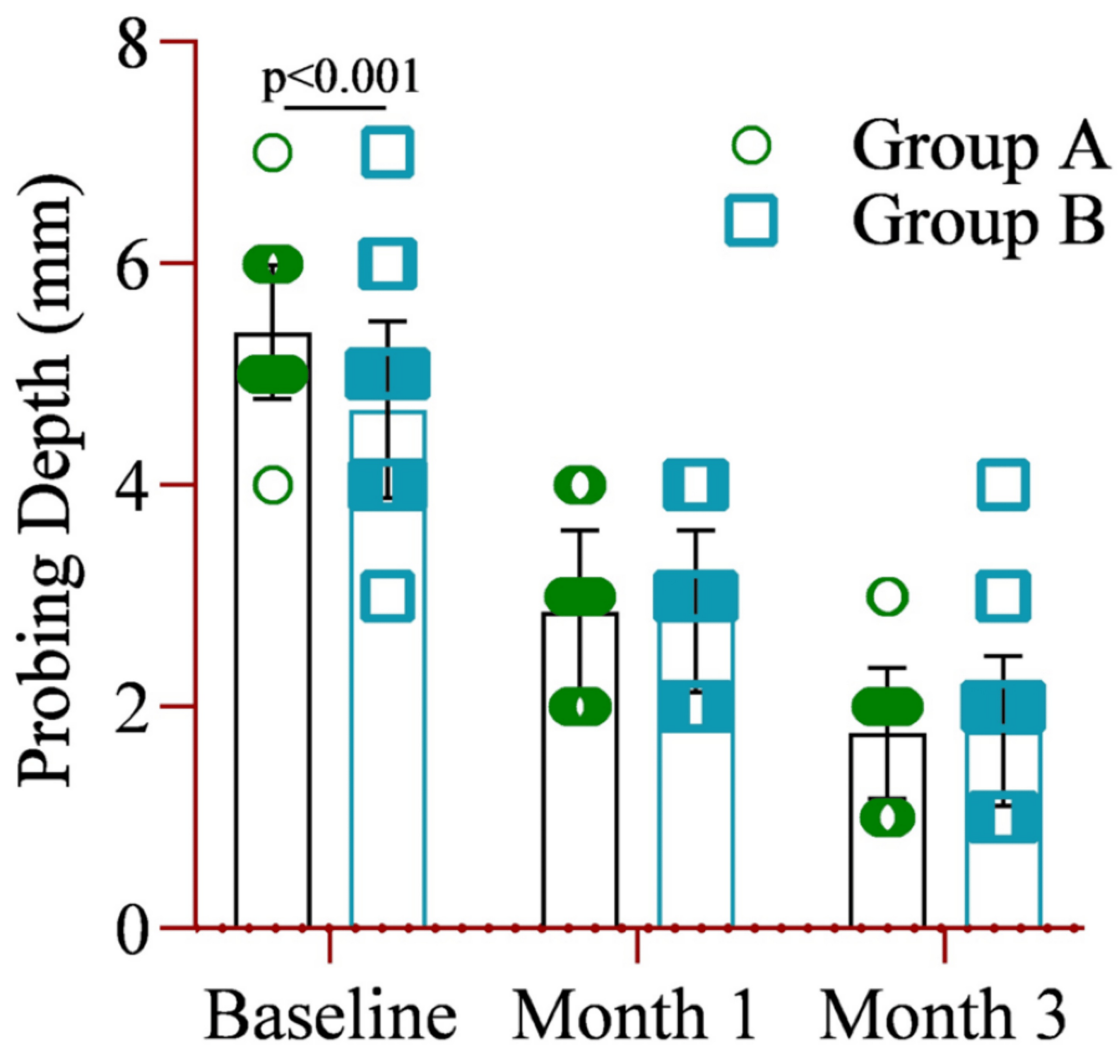
The mean difference in the probing index between Group A (amoxicillin and metronidazole) and Group B (probiotics placebo) at baseline, month one, and month three. Notes: Two-way ANOVA was used to estimate the p-value. Illustration Credit: Md Ahsanul Haq

Comparison of the mean probing index revealed that Group A had a significantly higher index (5.38±0.60) than Group B (4.68±0.79) at baseline. However, no significant differences were observed between the groups at months one and three (Figure 4).

**Figure 5:**
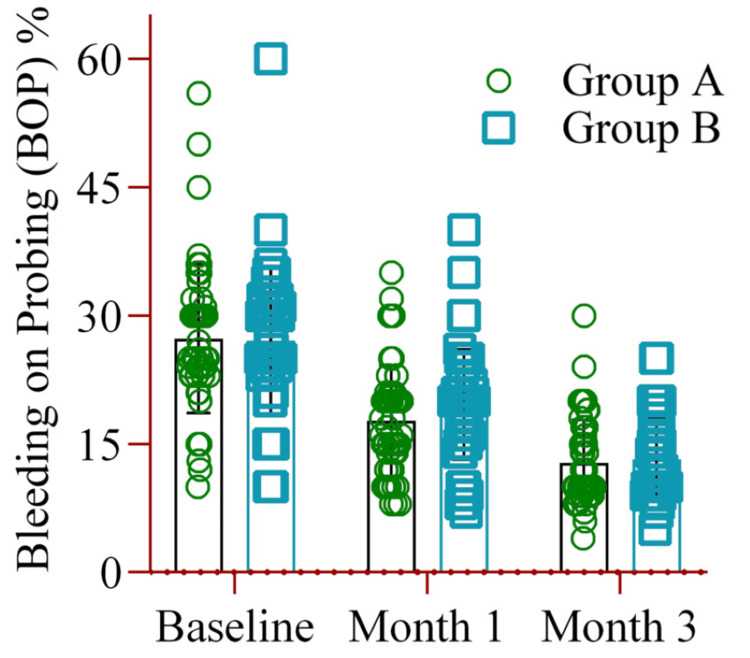
The mean difference in bleeding on probing (BOP %) between Group A (amoxicillin and metronidazole) and Group B (probiotics placebo) at baseline, months one, and month three. Notes: Two-way ANOVA was used to estimate the p-value. Illustration Credit: Md Ahsanul Haq

Bleeding on the PI was not significantly different between the two groups at baseline and during follow-up (Figure 5).

**Figure 6:**
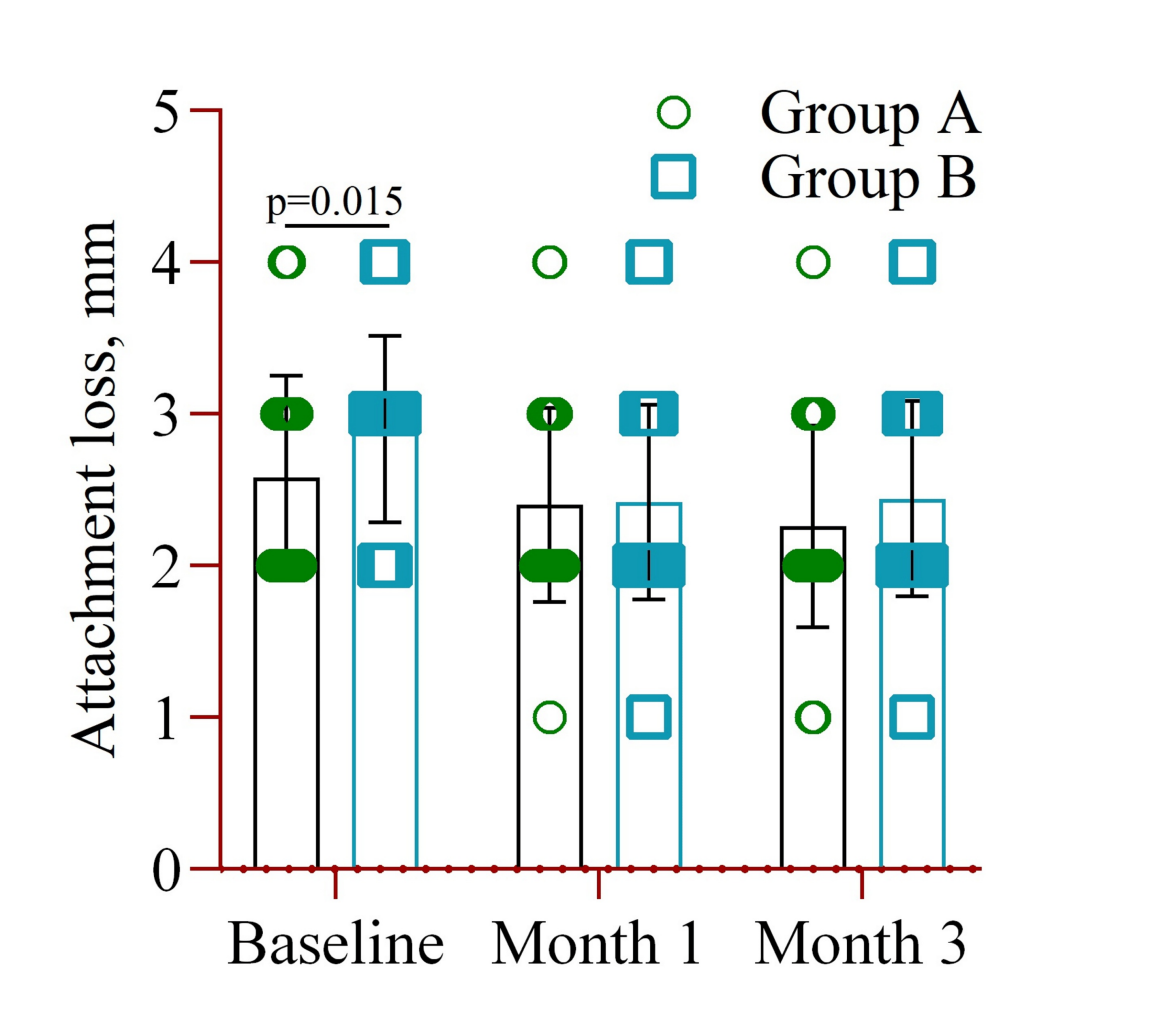
The mean difference in attachment loss (mm) between Group A (amoxicillin and metronidazole) and Group B (probiotics placebo) at baseline, month one, and month three. Notes: Two-way ANOVA was used to estimate the p-value. Illustration Credit: Md Ahsanul Haq

At baseline, Group A exhibited significantly lower attachment loss (2.58±0.67) compared to Group B (2.90±0.61) (p=0.015). However, no significant differences were observed between the groups at the follow-up visits at months one and three (Figure 6).

The results of the above analysis revealed significant differences at baseline in GI, PD, and attachment loss, suggesting potential selection bias during participant recruitment. The generalized estimating equations (GEE) model can address these baseline differences by incorporating covariates, providing an unbiased estimate of the treatment effect. Using the GEE model, it was observed that, during the study period, Group B showed a significant 23% increase in the GI (95% CI: 0.08-0.38; p=0.003) compared to Group A. In contrast, PD significantly decreased by 23% (95% CI: -0.38 to -0.07; p=0.004) in Group B compared to that in Group A (Table 1).

**Table 1:** Longitudinal changes in Group B (probiotics placebo) compared to Group A (amoxicillin and metronidazole) in the plaque index, gingival index, probing depth, bleeding on probing, and attachment loss during the study period. Notes: Data is presented as a beta coefficient with 95% confidence intervals in parentheses and adjusted for age, sex, duration of treatment, and history of contact with active TB cases. Statistical analysis was performed using the generalized estimating equation (GEE) model. The p-value of <0.05 is significant.

Parameters	β-coefficient (95% CI)	p-value
Group B (Probiotics)	-	-
Plaque index	0.04 (-0.17, 0.25)	0.717
Gingival index	0.23 (0.08, 0.38)	0.003
Probing depth, mm	-0.23 (-0.38, -0.07)	0.004
Bleeding on probing, %	0.83 (-1.50, 3.17)	0.483
Attachment loss, mm	0.16 (-0.07, 0.40)	0.167

## Discussion

As shown in the result, the probiotic group (*L. reuteri*) started with worse periodontal health at baseline (as indicated by statistically significant differences in parameters such as loss of attachment). The fact that there were no significant differences between the antibiotics (AMX and MTZ: amoxicillin and metronidazole) and probiotic groups at the one-month and three-month follow-ups suggests that the probiotic group experienced substantial improvement. This improvement was sufficient to bring their periodontal health on par with the antibiotic group, which started with better baseline periodontal health.

Both the treatment modalities evaluated in this study reduced PD in moderate pockets. However, this decline was statistically insignificant, probably due to the undesirable effects of smoking [24]. Various probiotics have been tested in animal [25,26] and human [12,27] trials to investigate their advantages in treating periodontal disease.

The study conducted by Teughels et al. [23] yielded similar findings, indicating no statistically significant difference in the pocket depth reduction between scaling and root planing and their association with *L. reuteri *in systemically healthy non-smokers. Nevertheless, the current study found a noteworthy decrease in the deep pockets' average pocket depth after 90 days in the probiotic group, compared to the initial measurements.

According to scientific evidence, it is pertinent to note that patients without altering circumstances experience a mean reduction of 1.29 mm in moderate pockets and 2.16 mm in deep pockets concerning PD [28]. In the current study, the AMX+MET group experienced a decrease of 1.76 mm in the average PD of deep pockets, whereas the *L. reuteri *group had a reduction of 2.34 mm. However, Group 1 still had slightly higher PD than Group 2, indicating no statistically significant difference between the two groups.

Other adjunctive therapies in smokers demonstrated a reduction in PD by 1.88 mm in deep pockets with the systemic administration of MTZ in conjunction with AMX over seven days [29] and 2.5 mm with multiple applications of photodynamic therapy (PDT) [29]. Local antibiotics reduced PD by 1.78 mm at 90 days, while the control group showed a 1.12 mm reduction [30].

No statistically significant difference in CAL was found between moderate and deep pockets across the entire cohort. Multiple studies have confirmed that smokers have a less favorable response to both non-surgical and surgical periodontal treatment [31-33]. A recent study showed that cigarettes and their metabolites impair neutrophil activation, disrupting functions and slowing their speed and direction [34]. Changes in the immune response of smokers may contribute to the onset and progression of severe periodontitis, increasing the risk of developing this oral condition disease.

Cigarettes alter the composition of the oral microflora by reducing the amount of oxygen available [35]. This is caused by the continuous release of nicotine and its byproducts, which harm the cellular metabolism of fibroblasts [36]. Additionally, cigarettes have an impact on both the humoral-mediated and cell-mediated immune responses of the host. All clinical studies on periodontal treatment with *L. reuteri* included patients without risk factors [22]. This differs from the current study, which aimed to evaluate smoking patients' clinical response to additional treatment probiotics.

*L. reuteri* produces reutericyclin, reuterin, and bacteriocins, which can hinder the growth of many infections [37]. This helps maintain a healthy balance of microorganisms in the microbiota of those who smoke. After periodontal treatment, *L. reuteri *shows enhanced anti-inflammatory properties by reducing TNF-α, IL-1β, and IL-17 levels in the gingival crevicular fluid [38].

The existence of residual pockets, defined as areas with a PD of 5 mm or more and bleeding after probing, indicates an unfavorable outcome of the treatment. These features signify the persistence of periodontal disease, which increases the likelihood of its advancement and ultimately necessitates further periodontal retreatment [19]. The study's findings showed a noteworthy decrease in the proportion of sites with periodontal disease measuring 5 mL or more and BOP in both groups.

Since probiotics pose a limited risk of developing illnesses, any clinical adverse effects must be documented [39]. The individuals in the current study did not report any negative health consequences. All 28 patients involved in the study were highly compliant. Regrettably, eight patients had to be excluded owing to various reasons. Initially, specific individuals had been gone for an extended duration, rendering them unable to participate in subsequent visits.

Furthermore, the study specifically targeted individuals in good health, both males and females. Patients who no longer met these criteria were regularly excluded from the study. Furthermore, patients with post-COVID syndrome no longer met the criterion for being in a healthy state. According to the criterion, physical and neuropsychiatric symptoms continue for more than 12 weeks without any other plausible explanation [40]. Although there was an improvement in all the parameters from baseline to three-month follow-up, both treatment groups are beneficial for improving periodontal health with the non-surgical therapy.

Limitations of the study

A potential limitation of the study is the lack of evaluation of the colonization of *L. reuteri. *If this factor had been assessed, more accurate results may have been produced. Since probiotics might have therapeutic effects in the gastrointestinal system without colonization or viability, the *L. reuteri *strains were not detected or quantified in this study. Furthermore, conducting such analysis would not have altered the findings of this study. A larger sample size yields more accurate outcomes.

## Conclusions

It may be inferred that the probiotic treatment successfully enhanced periodontal parameters to a degree similar to the antibiotic treatment, despite the probiotic group initially having more severe periodontal problems. This discovery provides evidence for the effectiveness of probiotics as a supplementary treatment to nonsurgical periodontal therapy, specifically in individuals who smoke and have widespread periodontitis.
